# SCOP/PHLPP1β mediates circadian regulation of long-term recognition memory

**DOI:** 10.1038/ncomms12926

**Published:** 2016-09-30

**Authors:** Kimiko Shimizu, Yodai Kobayashi, Erika Nakatsuji, Maya Yamazaki, Shigeki Shimba, Kenji Sakimura, Yoshitaka Fukada

**Affiliations:** 1 Department of Biological Sciences, Graduate School of Science, The University of Tokyo, Tokyo 113-0033, Japan; 2 Department of Cellular Neurobiology, Brain Research Institute, Niigata University, Niigata 951-8585, Japan; 3 Department of Health Science, School of Pharmacology, Nihon University, Chiba 274-8555, Japan

## Abstract

Learning and memory depend on the time of day in various organisms, but it is not clear whether and how the circadian clock regulates memory performance. Here we show that consolidation of long-term recognition memory is a circadian-regulated process, which is blunted by disruption of the hippocampal clock. We focused on SCOP, a key molecule regulating hippocampus-dependent long-term memory for objects. The amounts of SCOP and its binding partner K-Ras in the hippocampal membrane rafts exhibit robust circadian changes, and SCOP knockdown in the hippocampal CA1 impairs long-term memory at night. Circadian changes in stimulus-dependent activation of ERK in the hippocampal neurons are dependent on the SCOP levels in the membrane rafts, while *Scop* knockout abrogates the activation rhythm. We conclude that long-term memory formation is regulated by the circadian clock through SCOP dynamics in the membrane rafts of the hippocampal CA1.

## Abstract (web summary)

Learning and memory are subject to circadian variation, though the molecular mechanisms behind this are unclear. Here, the authors show SCOP, a regulator of hippocampal memory, undergoes circadian changes in CA1 membrane raft dynamics and contributes to time-dependent changes in long-term memory.

## Graphical abstract

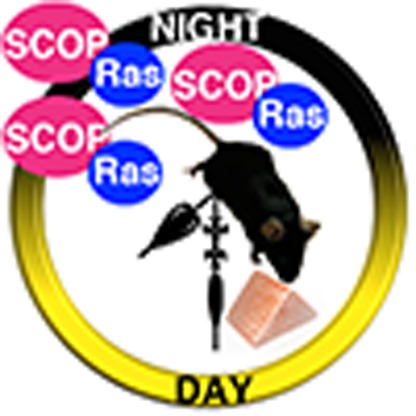

Physiological and behavioural rhythms with circadian periodicities are generated by cell autonomous circadian clocks that are synchronized to daily environmental changes such as the light–dark (LD) cycle. In mammals, the master circadian clock located in the hypothalamic suprachiasmatic nucleus (SCN) is synchronized to the LD cycle and governs the behavioural rhythms. On the other hand, peripheral clocks exist in most peripheral tissues, including extra-SCN brain regions. These clocks are regulated by neuronal and hormonal signals from the SCN^1^,^2^ and are thought to play key roles for physiological functions of the peripheral tissues. In the mouse hippocampus, expression levels of clock genes show circadian variations, supporting the presence of the peripheral clock regulated by the SCN^3^,^4^.

The circadian clocks regulate a variety of neural functions, including cognitive performance. In non-mammals, such as the zebrafish^5^, fruit fly^6^, cockroach^7^ and Aplysia^8^, circadian fluctuations in learning and/or memory have been observed under constant dark (DD) conditions, while their peaking times in the performance diverge among the organisms and/or the experimental paradigms. In humans^9^,^10^ and rodents^11^,^12^,^13^,^14^,^15^,^16^,^17^,^18^,^19^,^20^, several studies in LD cycles have demonstrated diurnal modulation of learning/memory performance on the Morris water maze task^11^,^12^, the novel location recognition task^13^, novel object recognition task^14^,^15^,^16^ and fear-related tasks^17^,^18^,^19^,^20^. Most of these studies in mammals have shown time-of-day-dependent variations under LD conditions, of which only two reports have addressed the circadian changes in fear-related memory performance under DD conditions^17^,^19^. To date, it is not known whether recognition memory shows a circadian variation and when it peaks in the day under constant conditions. Meanwhile, several studies in mice have demonstrated impairment on multiple memory tasks by lesioning of the SCN^21^ or disruption of the clock genes such as *Npas2* (ref. 22), *Period1* (ref. 23), *Cry1/Cry2* (ref. 24) and *Bmal*^25^. However, circadian rhythms of the memories have not been addressed in these mice. These studies suggest that the circadian clock is associated with memory performance, but a question remains as to whether and how recognition memory is controlled by the clock.

Spatial and declarative memories are processed in the hippocampus, especially in the area CA1 (refs 26, 27), in which Ras–ERK/MAPK pathway and the downstream CREB–cAMP response element transcriptional pathway play a pivotal role^28^,^29^,^30^,^31^. The extracellular signal-regulated kinases (ERKs) are activated by training for hippocampus-dependent memory, while hippocampal administration of ERK cascade inhibitors blocks CREB-cAMP response element-mediated transcription and contextual fear memory^28^,^32^,^33^. The mitogen-activated protein/extracellular signal-regulated kinase (MEK) inhibition in heterozygous *K-Ras* knockout (KO) mice disrupt long-term potentiation and hippocampus-dependent long-term memory^34^. These studies indicate that the activation of the K-Ras–ERK–CREB pathway is required for the regulation of hippocampus-dependent long-term memory. It is reported that ERK and CREB activities show daily (basal) fluctuations in the mouse hippocampus^19^, but a more important question remains unanswered as to whether training-induced ERK activation is under circadian control.

SCOP, suprachiasmatic nucleus circadian oscillatory protein, was originally identified as a molecule whose expression is circadian regulated in the rat SCN^35^ (later termed PHLPP1β (refs 36, 37)). SCOP protein is predominantly expressed in the central nervous system^35^, and particularly enriched in the hippocampus pyramidal cells from CA1 to CA3 (ref. 29), the brain areas important for memory formation^26^,^27^. SCOP directly interacts with the nucleotide-free form of K-Ras in the membrane rafts^38^, thereby inhibiting K-Ras function and its downstream ERK–CREB pathway in unstimulated hippocampal neurons^29^. On stimulation, SCOP is rapidly degraded by calpain that is activated by Ca^2+^-influx in response to brain-derived neurotrophic factor (BDNF), KCl or N-methyl-D-aspartate (NMDA) treatment in cultured neurons, or to training for a hippocampus-dependent memory task^29^. SCOP degradation in the hippocampus is critical for the activation of the K-Ras–ERK–CREB pathway and consequent memory formation^29^. SCOP degradation also controls the magnitude of long-term potentiation in hippocampal CA1 slice^39^. These findings raise the possibility that SCOP in the hippocampal neurons serves as a key mediator that links memory formation with the circadian clockwork.

## Results and Discussion

### Circadian regulation of long-term object recognition memory

**Figure 1:**
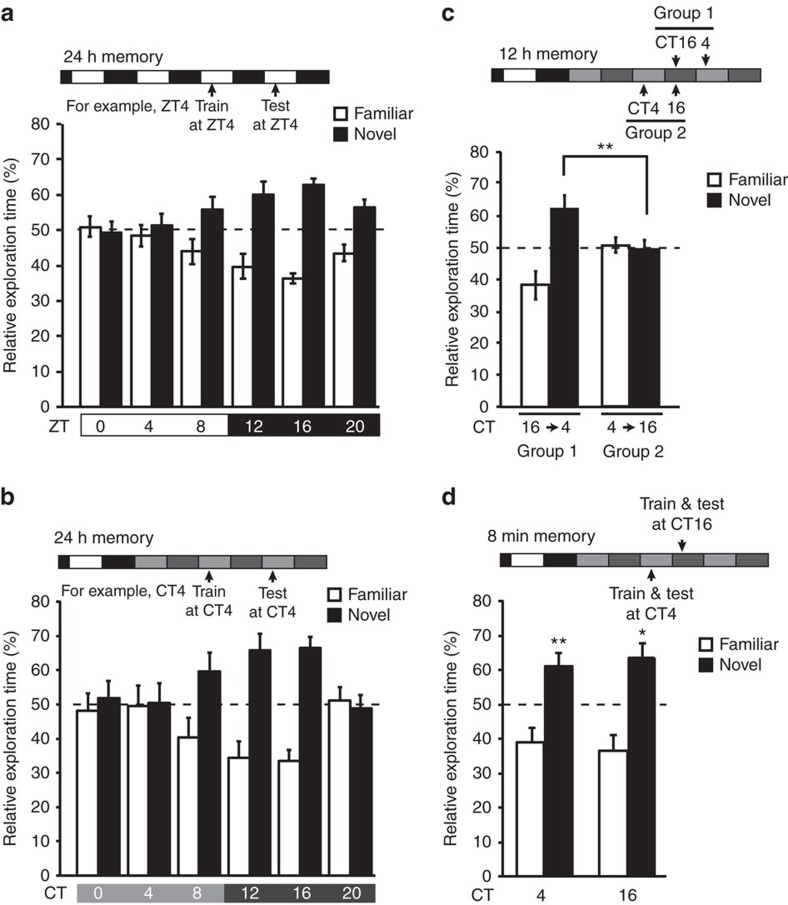
Daily and circadian changes in memory formation for object recognition. Mice entrained to a 12L/12D cycle were kept in the LD condition (**a**) or transferred to constant dim light (**b**–**d**) for the test of memory formation at various time points of the day. (**a**,**b**) Long-term memory performance was examined 24 h after training. *P*=0.014 in **a** and 0.034 in **b** by one-way analysis of variance. (**c**) Memory performance was also examined 12 h after training. The left pair (group 1) is the data from mice that were trained at CT16 and tested 12 h later (at CT4 in the next day), and the right pair (group 2) from mice that were trained at CT4 and tested 12 h later (at CT16). **P=0.0098 by Student's *t*-test. (**d**) Short-term memory performance was examined 8 min after training. **P=0.0043 (CT4) and *P=0.018 (CT16) by Student's *t*-test (versus 50%). Error bars, s.e.m. (**a**,**b**,**d**, *n*=8 mice; **c**, *n*=10). The dotted line represents performance by chance 50%.

We explored daily changes in hippocampus-dependent recognition memory of mice by examining the performance on a novel object recognition task. Male C57BL/6 mice were entrained to a 12 h light/12 h dark (LD) cycle and transferred to an experimental arena (Supplementary Fig. 1a) 5 min before the training (5 min) and testing (5 min) under a very dim light condition (4 lux), which enabled video recordings of mice behaviours and allowed mice to watch the objects. We measured the time periods (s) when the mice explored the familiar and novel objects during 5 min testing period 24 h after the training. Memory for the familiar object was assessed by the per cent ratio of the time spent for exploring the novel object versus total exploration time (towards the two objects). The training and testing were performed at various times of the day (8 mice for each time point). Long-term memory formation revealed a clear daily change with the peak during the early night at ZT16 (Fig. 1a), where ZT is zeitgeber time (ZT0 is defined as lights-on and ZT12 as lights-off).

We then examined potential circadian regulation of long-term memory formation by the internal clock. To this end, LD-entrained mice were kept under a constant dim light (4 lux) condition for 24 h (day 0), and then subjected to the long-term memory task under the 4 lux condition (Supplementary Fig. 1b). We found a robust circadian rhythm in long-term memory formation peaking at early night, that is, projected circadian time (CT) 16 (where, by convention, CT0 is defined as the lights-on time and CT12 as the lights-off time in the previous LD cycle; Fig. 1b). No significant variation was observed in total exploration time across the day (Supplementary Fig. 1c). To examine which of the training or the testing timing is important, we performed a 12 h novel object recognition task by testing memory formation 12 h after the training. The mice that were trained at CT16 and tested at CT4 (group 1) showed significantly higher memory performance than those trained at CT4 and tested at CT16 (group 2) (Fig. 1c). Together, the long-term memory performance depends on the timing of the training (peaking at early night), but not of the testing. Long-term memory is defined as a process comprising three stages: acquisition of new information; consolidation of the acquired information; and retrieval of stored information^40^. Our results revealed the importance of the timing of acquisition and/or consolidation stage for the circadian regulated memory performance. Because short-term memory (8 min memory task) was comparable between CT4 and CT16 (Fig. 1d), it is less likely that the circadian clock regulates the acquisition stage, which is shared by short-term and long-term memory formation^40^. Collectively, it was demonstrated that the circadian clock facilitates the consolidation process with the peak at early night in long-term memory formation.

### SCN clock and hippocampal clock regulate long-term memory

**Figure 2:**
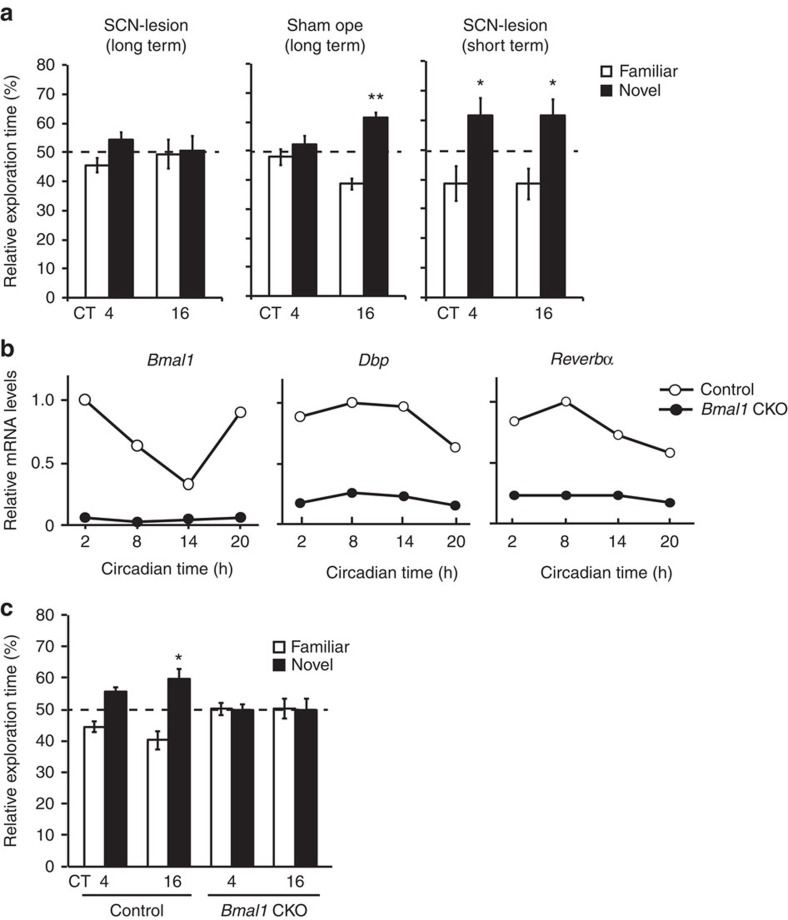
The SCN clock and hippocampal clock regulate long-term memory. (**a**) Long-term memory formation (24 h memory task) at CT16 (middle) was largely impaired by the SCN lesion (left). Error bars, s.e.m. (SCN lesion/long-term, *n*=12 mice; sham ope, *n*=11; short-term, *n*=8). **P=4.6E-5 (sham ope/CT16), *P=0.040 (SCN lesion/short-term/CT4) and *P=0.023 (SCN-lesion/short-term/CT16) by Student's *t*-test (versus 50%). (**b**) Temporal changes in *Bmal1*, *Dbp*, *Reverbα* mRNA levels in the hippocampal CA1 of *Bmal1* CKO (*Bmal1*^flox/flox^*;Emx1*^cre/+^) or control mice (*Bmal1*^flox/flox^*;Emx1*^+/+^). mRNA levels were quantitated by real-time RT–PCR. Three pairs of hippocampi were pooled and used for quantification at each time point. (**c**) Memory performance of the *Bmal1* CKO mice and their littermate control mice in the novel object recognition task. Error bars, s.e.m. (control, *n*=11 mice; *Bmal1* CKO, *n*=11). *P=0.026 by Student's *t*-test (versus 50%).

A role of the master circadian clock in long-term memory formation was explored by lesioning the SCN, which caused arrhythmic locomotor activities in constant dark (DD) condition^41^ (Supplementary Fig. 2a, upper panel). The SCN-lesioned mice failed to exhibit 24 h long-term memory formation both at CT4 and CT16 (Fig. 2a, left), whereas a sham operation had no significant effect on the circadian rhythmicity of the locomotor activity (Supplementary Fig. 2a, lower panel) or the circadian rhythmicity in long-term memory (Fig. 2a, middle). In contrast, short-term memory at both CT4 and CT16 was unaffected by the SCN lesioning (Fig. 2a right). These data demonstrate that the master circadian clock in the SCN governs the circadian rhythm of hippocampus-dependent long-term memory for object recognition.

We then investigated the importance of the peripheral clock in the hippocampus for long-term memory formation by generating *Bmal1* conditional KO mice (termed *Bmal1* CKO). *Bmal1* is an essential clock gene in the circadian clockwork^42^. We crossed *Bmal1* floxed mouse^43^ with *Emx1*^cre/+^ mouse^44^, which shows extensive Cre expression in the neocortex, hippocampus and olfactory bulb, with negligible expression in the other brain regions^44^. In the CKO mice, *Bmal1* mRNA levels in the hippocampal CA1 region were suppressed across the day, contrasting with the robust rhythm of *Bmal1* mRNA levels in the control (*Bmal1* floxed) mice CA1 (Fig. 2b). In consequence, expression of BMAL1 protein was largely reduced in the forebrain of *Bmal1* CKO mice (Supplementary Fig. 2b, full images of all the western blots are presented in Supplementary Fig. 7), and the mRNA levels of BMAL1-CLOCK-activated genes, *Dbp* and *Reverbα*, were both downregulated in the CA1 area of *Bmal1* CKO mice (Fig. 2b). The CKO mice showed circadian locomotor activity rhythms indistinguishable from those of wild-type (WT) mice (Supplementary Fig. 2c,d). Thus, *Bmal1* CKO caused dysfunction of hippocampal clock leaving the master SCN clock intact.

The long-term recognition memory of *Bmal1* CKO mice revealed a significant decline in the performance at CT16 (Fig. 2c), while the littermate control (*Bmal1* floxed, Fig. 2c) and *Emx1*^cre/+^ mice (Supplementary Fig. 2e) showed the circadian variation in the performance as seen in WT mice (Fig. 1b). These data suggest that the forebrain clock, probably the hippocampal clock, is responsible for the circadian variation in long-term memory for object recognition.

### Long-term memory rhythm is disrupted in *Scop*-deficient mice

**Figure 3:**
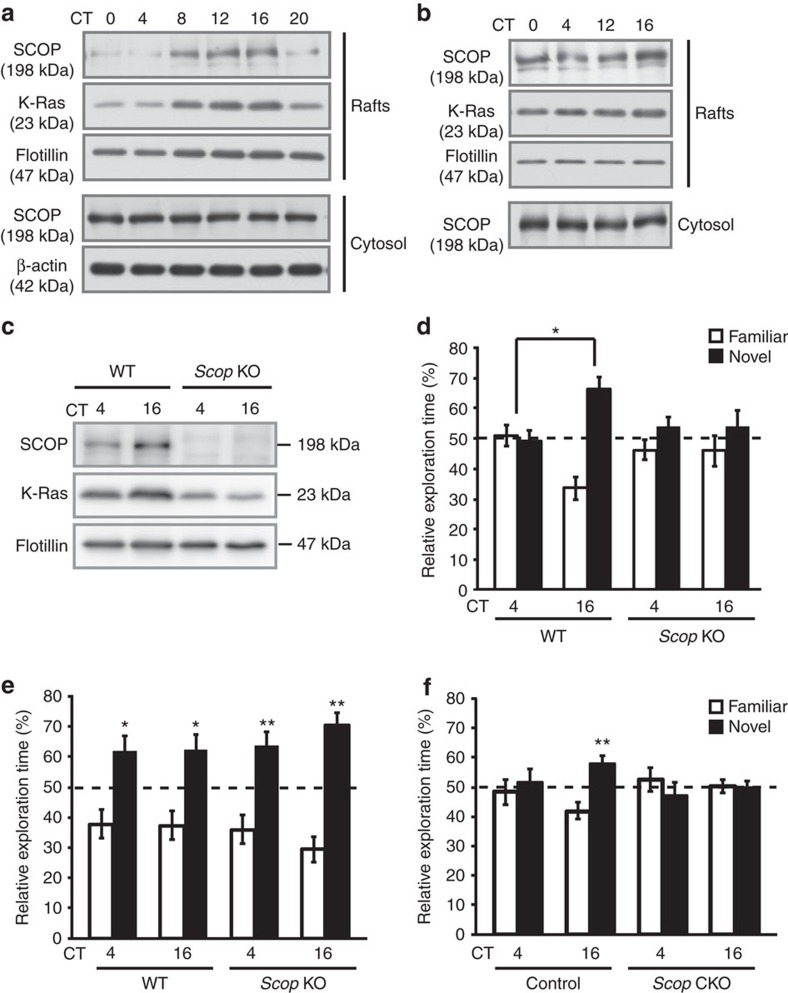
SCOP protein levels in hippocampal CA1 and the circadian variation in memory formation in *Scop* KO. (**a**,**b**) Temporal profiles of SCOP and K-Ras protein levels in the membrane rafts and the cytosol of the hippocampal CA1 (**a**) and the whole hippocampus (**b**) of mice reared under the constant dim light condition. Flotillin was used as a marker of the membrane rafts. Similar results were observed from *n*=3 biological replicates. (**c**) SCOP and K-Ras protein levels in the raft fraction of the hippocampal CA1 of WT or *Scop* KO hippocampus at CT4 and CT16 under constant dim light condition. Similar results were observed from *n*=2 biological replicates. Immunoblot images in **a**–**c** have been cropped for presentation. Full images of the blots are shown in Supplementary Fig. 7. (**d**) Long-term 24 h memory performance of *Scop* KO and WT mice in the novel object recognition task. *P=0.030 (WT) by Student's *t*-test. *P*=0.12 in *Scop* KO (CT4 versus 16) by Student's *t*-test. (**e**) Short-term memory formation assessed by 8 min memory task in *Scop* KO and WT mice. *P=0.018 (WT/CT4), 0.016 (WT/CT16), 0.0067 (*Scop* KO/CT4) and 4.9E-5 (*Scop* KO/CT16) by Student's *t*-test (versus 50%). Error bars, s.e.m. (long-term, *n*=11 mice; short-term, *n*=13 for *Scop* KO, *n*=11 for WT). (**f**) Long-term (24 hr) memory performance of *Scop* CKO (*Scop*^flox/flox^*; Emx1*^cre/+^) or control mice (*Scop*^flox/flox^*;Emx1*^+/+^) on the novel object recognition task. **P=0.0096 (control/CT16) by Student's *t*-test (versus 50%). *P*=0.91 (*Scop* CKO/CT16) by Student's *t*-test (versus 50%).Error bars, s.e.m. (*n*=10 mice for *Scop* CKO; *n*=8 for control).

To delineate the molecular mechanism underlying the circadian regulation of hippocampus-dependent long-term memory, we focused on membrane rafts where SCOP interacts with K-Ras^38^. Although no significant variations were observed for SCOP and K-Ras levels in the whole hippocampal rafts (Fig. 3b), the CA1 rafts showed robust circadian oscillations in the amount of the two proteins, both peaking at CT16 (Fig. 3a), a peaking time of long-term recognition memory (Fig. 1b). The circadian variation of SCOP in the rafts is contrasting with almost constant levels of cytosolic SCOP in the CA1 (Fig. 3a), which were ∼20-fold higher than those found in the rafts (Supplementary Fig. 3).

We investigated the role of SCOP in the circadian regulation of long-term memory formation by producing *Scop* deficient (KO) mice (Supplementary Fig. 4a,c,d). The KO mice were viable, fertile and apparently indistinguishable from WT mice. SCOP protein was undetectable in the hippocampal lysate of the KO mice (Supplementary Fig. 4g), and no immunoreactivities were detected in the pyramidal cell layer of the hippocampal sections (Supplementary Fig. 4h). In locomotor activity recordings, no significant difference was observed in the circadian period in DD between *Scop* KO and WT (Supplementary Fig. 4i) as reported^45^, indicating that *Scop* deficiency had no discernible effect on the SCN.

We found that *Scop* deficiency reduced K-Ras levels in the hippocampal CA1 rafts at both CT4 and CT16, and blunted the circadian variation (Fig. 3c). These observations suggest that a large proportion of K-Ras within the CA1 rafts is sequestered by SCOP in a rhythmic manner. *Scop* deficiency significantly diminished long-term (24 hr) memory formation at CT16 to a level comparable to that at CT4 (Fig. 3d), indicating that *Scop* is indispensable for rhythmic formation of long-term recognition memory. On the other hand, *Scop* deficiency had no significant effect on 8 min short-term memory (Fig. 3e). The impact of *Scop* deficiency in the hippocampus was examined by generating conditional *Scop* KO (termed *Scop* CKO) mice (Supplementary Fig. 3b,e,f, see Methods) by crossing *Scop* floxed mouse with *Emx1*^cre/+^ mouse^44^. In the hippocampal lysate of the CKO mice, SCOP protein was almost undetectable (Supplementary Fig. 4g). *Scop* CKO mice failed to show long-term (24 h) memory performance at CT16, whereas the littermate control (*Scop* floxed) mice showed higher memory performance at CT16 than at CT4 (Fig. 3f). Because *Scop* deficiency reduced K-Ras levels in the membrane rafts and blunted their rhythmicity (Fig. 3c), it is probable that the circadian variation in K-Ras associated with SCOP in the membrane rafts confers circadian rhythmicity on long-term memory formation.

### SCOP knockdown in CA1 abolished long-term memory at night

**Figure 4:**
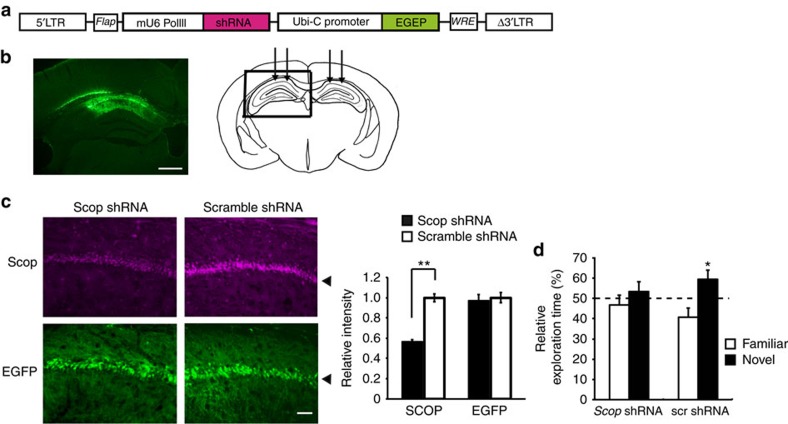
Effect of SCOP knockdown in CA1 on long-term memory at night. (**a**) Schematic diagram of the shRNA-expressing lentiviral vector. The shRNA is under the control of mouse *U6-RNA Pol III* promoter (mU6 Pol III), and a human *UbiC* promoter drives EGFP marker gene for tracking transduced cells. 5′-LTR, HIV-1 5′-LTR; Δ3′ LTR, HIV-1 self-inactivating 3′-LTR; *Flap*, HIV-1 DNA flap element; *WRE*, woodchuck hepatitis B virus RNA regulatory element. (**b**) A representative EGFP fluorescence image of a coronal section of the hippocampus of mice that received shRNA lentivirus. The diagram (right) identifies the hippocampal area shown in the photograph (left). Arrows indicate four injection points of shRNA lentivirus. Scale bar, 500 μm. (**c**) SCOP immunoreactivities (upper panels) and EGFP fluorescence images (lower panels) in the hippocampal CA1 region transduced with lentivirus expressing *Scop* shRNA (left panels) or control scr shRNA (right panels). Arrowheads indicate the pyramidal cell layer. Scale bar, 50 μm. The average intensity of immunofluorescence was quantified from four randomly selected areas in the pyramidal cell layer (bar graph). The average of scr shRNA was set at 1.0. **P=9.3E-5 by Student's *t*-test. (**d**) Long-term (24 hr) memory performance at CT16 in mice that received lentivirus expressing *Scop* shRNA or scr shRNA. *P=0.032 (scr shRNA) and *P*=0.24 (*Scop* shRNA) by Student's *t*-test (versus 50%). Error bars, s.e.m. (*n*=10 mice).

To narrow down the region responsible for *Scop*-mediated circadian variation in long-term memory, *Scop* in the hippocampal CA1 was knocked down by injecting a lentiviral vector expressing *Scop* shRNA (a short hairpin RNA targeting *Scop*) and enhanced green fluorescent protein (Fig. 4a). In NIH3T3 cells, infection of the *Scop* shRNA lentivirus efficiently reduced the level of endogenous SCOP, while that of a scrambled shRNA (scr shRNA) lentivirus had no significant effect on SCOP expression (Supplementary Fig. 5). Two parallel injections were given to each unilateral CA1 area (four injections per animal) to cover the whole CA1 area (Fig. 4b), where the *Scop* shRNA lentivirus significantly reduced SCOP immunoreactivities (Fig. 4c). The mice were allowed to recover from the surgery for at least 2 weeks, and then the long-term recognition memory test was performed at CT16, which is the peak time of the memory performance in WT mice (Fig. 1b). *Scop* knockdown in the CA1 suppressed the preference for the novel object, contrasting to the control experiment with the lentivirus expressing scr shRNA (Fig. 4d). These results indicate that SCOP protein in the hippocampal CA1 is indispensable for the long-term recognition memory at night.

### Circadian variation of ERK activation in hippocampal neurons

**Figure 5:**
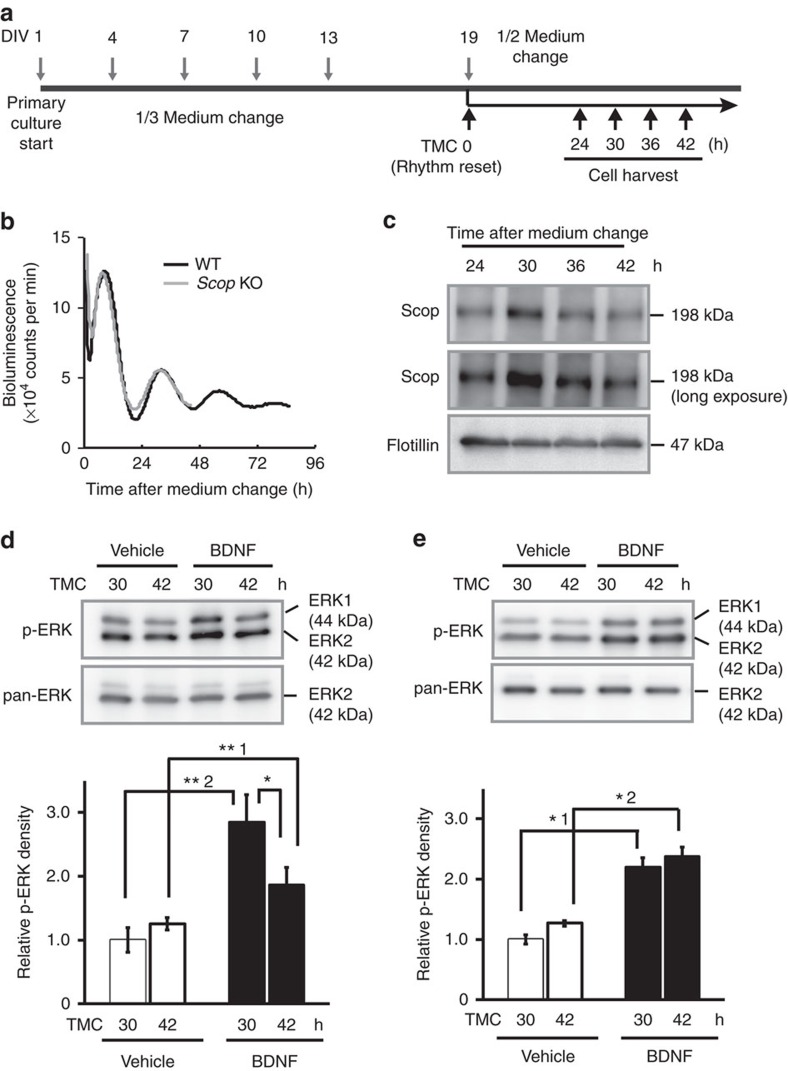
Circadian change in stimulus-induced ERK activation depends on SCOP levels in the rafts of cultured hippocampal neurons. (**a**) A diagram of experimental schedule for primary culture of hippocampal neurons. Hippocampal neurons were isolated at days *in vitro* (DIV) 1. The neurons were maintained with changes of one-third of the medium to a fresh medium every 3 days. Bioluminescence rhythms were reset by replacing half of the medium with a fresh medium at DIV 19 after 6-day culture without medium change from DIV 13. TMC 0 was set at the time point of the half medium change. Cells were collected at TMC24, 30, 36 and 42. (**b**) A representative recording of the bioluminescence signals from the cultured neurons. The hippocampal neurons were prepared from newborns of WT (black line) or *Scop* KO (glay line) PER2::LUC mice. Similar results were observed from *n*=5 biological replicates. (**c**) Temporal change in SCOP protein levels in the membrane rafts of the hippocampal neurons in culture. Flotillin is a marker of the membrane rafts. Hippocampal neurons were pooled from 10 plates of 35 mm dish. Similar results were observed from *n*=3 biological replicates. (**d**) Representative western blotting data of pERKs and ERK in cultured hippocampal neurons after 15 min BDNF treatment (upper panels) and the quantitated data from three independent experiments (bottom). *n*=3 biological replicates. **^1^*P*=0.005, **^2^*P*=0.006 and *P=0.047 by Student's *t*-test. (**e**) Representative western blotting data of pERKs and ERK in *Scop* KO hippocampal neurons after 15 min BDNF treatment (upper panels) and their quantitated data (bottom). *n*=3 biological replicates. *^1^*P*=0.043 and *^2^*P*=0.045 by Student's *t*-test. *P*=0.37 in BDNF (TMC30 versus 42) by Student's *t*-test. The band densities of pERKs were shown as values normalized to ERK2 band density. The data from three independent experiments were expressed as the mean±s.e. The average of the protein levels at TMC30 h of vehicle control was set at 1.0. Immunoblot images in **c**–**e** have been cropped for presentation. Full images of the blots are shown in Supplementary Fig. 7.

We investigated how SCOP levels in the membrane rafts regulate the activation of ERK pathway in cultured hippocampal neurons isolated from PER2::LUC knock-in newborn mice^46^. After maturation of the neurons in culture, a half-medium change on days *in vitro* 19 reproducibly induced a robust circadian rhythm of the bioluminescence signals (Fig. 5a,b). Under this condition, we detected a circadian variation in SCOP levels in the membrane rafts of the cultured neurons showing a peak at time (h) after the medium change (TMC) 30 and a trough at TMC42 (Fig. 5c). At these two time points, we applied BDNF to induce ERK activation through Ca^2+^/calpain-dependent degradation of SCOP^29^. BDNF treatment at TMC30, when SCOP level was higher, activated (phosphorylated) ERKs more potently than that at TMC42 does (Fig. 5d). In *Scop* KO neurons, on the other hand, no significant difference was observed in BDNF-evoked ERK activation between the two time points (Fig. 5e), while their bioluminescence rhythms were indistinguishable from those of the WT (PER2::LUC knock-in) neurons (Fig. 5b, grey line). These observations demonstrate that the degrees of ERK activation vary parallel to the circadian change in SCOP levels in the membrane rafts of the cultured neurons. Because BDNF treatment induced SCOP degradation at both TMC30 and 42 (Supplementary Fig. 6a), it is probable that when the amount of SCOP is higher in the hippocampal rafts, SCOP degradation releases more K-Ras and hence activates more ERKs. Consistently, in the presence of cell-permeable calpain inhibitor III, BDNF-induced activation of ERKs was reduced and became comparable between TMC30 and 42 (Supplementary Fig. 6b).

### Importance of SCOP in circadian memory formation

**Figure 6:**
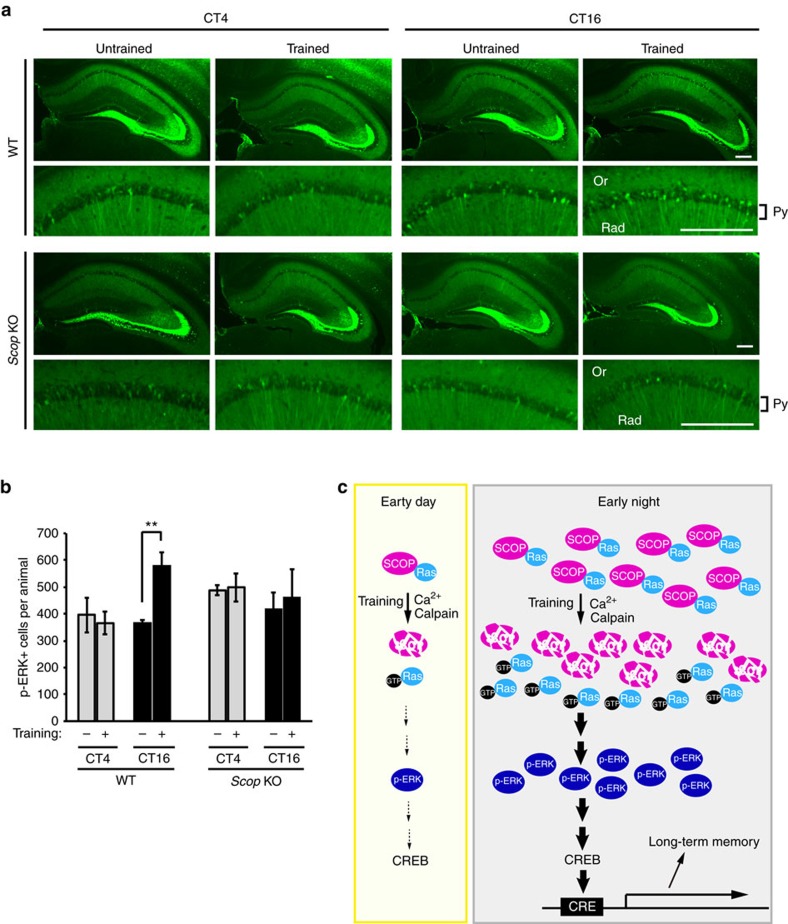
Circadian change in learning-dependent ERK activation *in vivo*. (**a**) Representative data of p-ERK immunoreactivities in coronal hippocampal sections (upper panels) and the magnified CA1 areas (lower panels) from WT and *Scop* KO mice. At CT4 or 16, p-ERKs were analysed by immunohistochemistry 5 min after 5 min training in the experimental arena with two objects. Untrained mice were similarly placed in the arena without objects. Scale bars, 300 μm. Or, oriens layer; Py, pyramidal layer; Rad, radiatum layer. (**b**) The number of p-ERK immuno-positive cells in CA1 pyramidal layer was integrated across total 18 (9 bilateral) hippocampal sections from each animal. These numbers were averaged across animals in each group. Data are mean±s.e.; *n*=4 mice for training (+) in WT at CT4 and 16, and *n*=3 for the other groups. **P=0.0091 by Student's *t*-test. *P*=0.74 (*Scop* KO/CT16, Training − versus +) by Student's *t*-test. (**c**) A model for circadian change in long-term memory formation. Learning activates ERKs in the CA1 more potently when SCOP level is higher in the membrane rafts.

We investigated whether learning-dependent activation of ERKs is regulated by the circadian-oscillating SCOP levels in the hippocampal membrane rafts *in vivo*, To estimate the learning-dependent ERK activation, phosphorylated and activated ERKs (p-ERK)-positive cells were quantified in hippocampal CA1 of mice that had been subjected to the training for novel object recognition at CT4 or 16. The number of p-ERK positive cells in the trained mice was significantly higher than that in untrained mice at CT16 (Fig. 6a,b). In contrast, the training at CT4 caused no detectable increase in p-ERK-positive cells (Fig. 6a,b). In *Scop* KO mice, the number of p-ERK-positive cells were unaffected by the training at both CT4 and CT16. It is concluded that the variation of SCOP levels in the CA1 rafts confers circadian rhythmicity on learning-induced activation of K-Ras–ERK pathway. Previously, the first author of this manuscript (K.S.) and co-workers demonstrated the training-induced activation of ERKs in the hippocampus and SCOP-mediated novel object recognition memory^29^. In that study, mice were trained and tested for objects only at ZT11-12 at which the training-dependent activation of ERKs was verified (the time information was not given in ref. 29), and the time (ZT11-12) was chosen because the author thought that the performance of nocturnal animals (mice) should be examined at a time close to the night time. The previous observations^29^ are not inconsistent with the present results that showed ERK activation in the CA1 after the training at CT16, but not at CT4, which is the time when mice do not learn well (Fig. 1b). It is probable that in LD cycle (Fig. 1a), ERK activation can be seen during ZT8-20 (when mice learn well) after the 5 min training for the novel object recognition task.

Here we emphasize two important properties of SCOP in neurons. First, in a quiescent state, SCOP can tether the nucleotide-free form of K-Ras in the membrane rafts (ref. 38 and Fig. 3c). Second, it is degraded in response to stimuli (such as learning) and it releases K-Ras, which is readily activated by GTP-binding^38^ and activates ERK–CREB pathway^29^. In the present study, we found that SCOP levels in the membrane rafts show a remarkable circadian variation in the hippocampal CA1 region, peaking at early night (Fig. 3a). It is conceivable that SCOP accumulated in the CA1 rafts retains more K-Ras in the early night. In fact, we found that BDNF treatment more potently activates ERKs when the amount of SCOP is higher in the hippocampal rafts in cultured neurons (Fig. 5d). Also, learning activates ERKs in the CA1 more potently when SCOP level is higher in the rafts (Fig. 6a,b). On the basis of the unique properties of SCOP, we propose a model (Fig. 6c) in which the circadian variation of SCOP levels in the hippocampal CA1 neurons produces the rhythm in long-term memory for object recognition with a peak in the early night. In nocturnal rodents, long-term potentiation observed in hippocampal slices was more enhanced in the night when compared with the daytime^47^,^48^, supporting our observation that learning at early night results in higher memory performance. For nocturnal animals, learning at night (that is, during their active phase) should be important in the wild because they have to learn and memorize a variety of foods and safe places during the active phase. In contextual or cued fear conditioning, on the other hand, mice display optimal memory formation during the (subjective) day^17^,^19^.These fear-based memories are mainly dependent on the amygdala, and it is conceivable that amygdala-dependent memory may have different kinetics of circadian oscillation than hippocampus-dependent recognition memory. Such a difference may be due to diverged phases of the clock oscillation between the amygdala and the hippocampus^49^ and/or a difference in mechanism connecting the clock and memory formation.

The loss of long-term memory seen with *Bmal1* CKO (Fig. 2c) was accompanied by hyperaccumulation of SCOP in the CA1 rafts (Supplementary Fig. 2f,g). It is noteworthy that transgenic mice overexpressing SCOP in the forebrain also lose long-term memory formation^29^. When SCOP accumulates abnormally in the hippocampus, Ca^2+^/calpain activated by the training may be unable to degrade sufficient amounts of SCOP for activation of K-Ras^29^, with the assumption that a large excess of SCOP over K-Ras is present in the CA1 rafts. In contrast, when SCOP was knocked down (Fig. 4) or knocked out (Supplementary Fig. 4g,h), K-Ras does not accumulate in the CA1 rafts (Fig. 3c), and the training cannot efficiently activate the signalling pathway for long-term memory formation (Figs 3d,f and 4d). These observations strongly suggest that SCOP levels in the membrane rafts need to be maintained within a physiologically appropriate range in order for the K-Ras–ERK–CREB pathway to be activated in a training-dependent manner.

SCOP is detected not only in the membrane rafts but also in non-raft membranes and cytosol^35^, among which only the rafts showed a significant rhythm in SCOP protein levels (Fig. 3a). It is proposed that the membrane rafts spatially organize signalling molecules and facilitate the signal transduction^50^. Our data together indicate that the dynamic variations of SCOP and K-Ras co-localized in the membrane rafts are particularly important for the circadian regulation of long-term memory. The mechanism of how an appropriate amount of SCOP is timely recruited to the membrane rafts is one of the important issues to be addressed in future studies.

## Methods

### Housing of mice

Male C57BL/6 mice at ages of 8–16 weeks were used for the experiments. Animals were reared under a LD cycle, in which food and water were available *ad libitum* under controlled temperature at 23 °C and humidity at 50%. All animal experiments were performed in accordance with the guidelines of The University of Tokyo.

### Mutant mice

*Emx1*^Cre/+^ knock-in mice were kindly provides by Dr Atsu Aiba (Graduate School of Medicine, the University of Tokyo)^44^. *Bmal1* floxed mice were generated by S.S.^43^.

### Generation of *Scop* KO mice

All procedures for the generation of *Scop* KO mouse were carried out in accordance with the guidelines laid down by the animal welfare committee and the ethics committee of Niigata University. *Scop*^flox/flox^ (*Scop*-floxed) mice were generated by introducing a mutation into the *Scop* locus with two *loxP* sequences flanking exon 4 of the *Scop* gene encoding the first leucine-rich repeat domain (Supplementary Fig. 4a). *Scop*-floxed mice were generated using homologous recombination in the C57BL/6N embryonic stem cell line RENKA^51^. The *Scop* gene was isolated by screening a bacterial artificial chromosome library prepared from the C57BL/6 strain (Incyte Genomics). A *Scop* targeting vector contained exon 4 of the *Scop* gene, 4.3 kb upstream and 7.78 kb downstream homologous genomic DNA fragments, and a diphtheria toxin gene for negative selection (Supplementary Fig. 4a). A DNA fragment carrying a 34 bp *loxP* sequence and *Pgk*-1 promoter-driven neomycin phosphotransferase gene (*Pgk*-neo) flanked by two Flp recognition target (*frt*) sites was inserted into the site 233 bp upstream of exon 4. Another loxP site was introduced into the site 223 bp downstream of exon 4 to eliminate the exon 4 after Cre-mediated recombination. Homologous recombinants were identified by Southern blot analysis under the following conditions: *Kpn* I-digested DNA hybridized with 5′ probe, 40 kb for WT and 12.5 kb for targeted allele; *Bgl* II-digested DNA hybridized with 3′ probe, 11.9 kb for WT and 13.7 kb for targeted allele; *Sac* I-digested DNA hybridized with neo probe, 11.5 kb for WT (Supplementary Fig. 4c). Embryonic stem cell clones with the desired recombination were used to yield chimeric mice as described previously^52^. Chimeric mice were mated with C57BL/6 mice to obtain *Scop*^flox/+; neo/+^ mice.

*Scop*^flox/*+*; neo/+^ mice were further crossed with *TLCN-Cre* mice^53^,^54^ to yield heterozygous KO mice (Supplementary Fig. 4a). Homozygous *Scop* KO mice were obtained by crossing heterozygous pairs. The genotype for subsequent breedings of *Scop* KO mice was determined by PCR using a specific primer set outside of *Scop* exon 4 (Supplementary Fig. 4d). PCR genotyping of mouse tail DNA was performed using following primers: forward, 5′-CTGTCTAGTGCATGGTTGTG-3′; reverse, 5′-ATGAGGCAGATTCTCCGAGC-3′.

To delete the neo gene from the targeted locus *in vivo*, *Scop*^flox/+; neo/+^ mice were mated to FLP66 transgenic mice of the C57BL/6 strain carrying a *Flp* recombinase gene under the control of the *EF1α* promoter^55^, and we obtained *Scop*^flox/+^ mice (Supplementary Fig. 4b). The elimination of the neo gene was confirmed by PCR analysis using the primer set: forward, 5′-CTGTCTAGTGCATGGTTGTG-3′; reverse, 5′-AAGGCTACAACAGGAGAGGT-3′ (Supplementary Fig. 4e). To generate forebrain-specific *Scop* KO mice (*Emx1*^cre/+^; *Scop*^flox/flox^, *Scop*-CKO), *Scop*^flox/+^ mice were bred with *Emx1*^Cre/+^ knock-in mice. The *Cre* gene was detected by PCR using the primer set: forward, 5′-ACCTGATGGACATGTTCAGG-3′; reverse, 5′-ACCAGGCCAGGTATCTCTG-3′ (Supplementary Fig. 4f).

### Locomotor activity rhythms

Mice were housed individually and their spontaneous locomotor activities were recorded using an area sensor (Elekit) with an infrared detector. Locomotor activity was collected every minute and analysed by ClockLab software (Actimetrics).

### Novel object recognition task

The novel object recognition task is a benchmark test of recognition memory in the rodent. This task is non-rewarded and fear-irrelevant test based on the innate tendency of rodents to seek novelty^26^, dependent on the integrity of the hippocampus^56^. In this study, mice were housed individually in their home cages, handled (∼2 min a day), and habituated to the testing arena (for 2 min) daily for at least 1 week before the behavioural task. Every day animals were handled at different times of the day to ensure that they did not entrain to handling by the experimenter at a specific time. For circadian tests, the animals were entrained to the LD cycle and then placed under constant dim white light condition from CT0 (ZT0) the day before the training for the novel object recognition task. The dim light (4 lux, 3.3 μW cm^−2^, at the bottom position of a mouse cage or an experimental arena) was provided by a wide-panel LED on the top of a light-tight compartment. The training and testing were carried out in the acrylic open arena (W22.0, D38.0, H19.5 cm) with a video camera mounted obliquely overhead. Before starting the training, mice were first habituated to the arena (4 lux) for 5 min. Then, two identically shaped objects were presented for 5 min (training), after which mice were reared in the home cage for 24 h. The mice were then transferred back to the same arena, where one of the original objects and a new object were presented for 5 min (testing) after 5 min habituation to the arena. During training and testing, the time spent exploring each object was recorded for 5 min. The time spent exploring these two objects were measured from the video recordings by observers blind to the experiment. The relative exploration time was expressed as a per cent time of the total exploration time that a mouse spent exploring the object. The total exploration time was set to 100% and the exploration times to the two objects were measured as the times for ‘familiar' and ‘novel'. Trials were excluded if a mouse spent <10 s for exploring both the objects. The objects used for this task differed in shape, but were the same in surface texture and colour. In preliminary experiments, we first examined whether mice showed no significant preference between any pair of objects (data not shown). The objects and the arena were washed between every trial to remove odour cues. In all behavioural experiments, mice were randomized according to age in weeks. The sample size required was estimated to be *n*=8–13 per group on the basis of previous studies examining memory performance in object recognition.

### SCN lesioning

Male C57BL/6 mice aged 8 weeks were anaesthetized by an intraperitonial injection (20 μl g^−1^ body weight) of a mixture of ketamine (7 mg ml^−1^, Daiichi Sankyo Propharma) and xylazine (0.44 mg ml^−1^, Bayer Health Care) dissolved in bacteriostatic saline. The anaesthetized animals were placed in a stereotaxic frame (Narishige Inc.) with both bregma and lambda lines at the horizontal level. Small burr holes were drilled in the skull according to the following coordinates: mediolateral (ML) ±0.23 mm; anteroposterior (AP) 0.2 mm; and dorsoventral (DV) 5.9 mm, and an electrode (a diameter of 100 μm) coated with epoxy (except for 200 μm at the tip) was moved downward to the target site. An electric current of 0.8 mA was delivered by a lesion-making device (Ugo Basile) for 3 s. This procedure was performed bilaterally. Sham operation controls were subjected to the identical surgical procedure but were delivered no current. The incisions were sutured and the animals were allowed to recover from the surgery. Free-moving activity rhythms were monitored under DD condition to confirm arrhythmicity induced by SCN lesioning. The animals were again entrained to the LD cycle for at least 2 weeks before starting the novel object recognition task. Behavioural data were analysed using ClockLab software (Actimetrics). The SCN lesion was confirmed by nissl staining of the SCN slices after the behavioural tests (Supplementary Fig. 2a, left), and any mice lesioned outside the SCN were eliminated from the data analysis.

### Western blot analysis

Proteins separated by SDS–PAGE were transferred to a polyvinylidene difluoride membrane (Millipore). The blot was blocked in a blocking solution of 3% (w/v) skim milk or 3% bovine serum albumin (BSA; for detection of SCOP) in T-TBS (0.05% Tween20, 50 mM Tris-HCl, 140 mM NaCl and 1 mM MgCl_2_; pH 7.4), for 2 h at room temperature. Then the blots were incubated for 4 h at room temperature with a primary antibody diluted in the blocking solution. The signals were visualized by an enhanced chemiluminescence detection system (PerkinElmer). Primary antibodies used were as follows: anti-SCOP (1:2,000, αEC or αCB, in Shimizu *et al*.^35^); anti-K-Ras (1:1,000, Santa Cruz Biotechnology, Inc., sc-30); anti-flotillin-1 (1:2,000, BD Transduction Laboratories #610820); anti-β-actin (1:5,000, Sigma, AC-74); p-ERK (1:1,000, Cell Signaling #9101); and pan-ERK (1:5,000, BD Transduction Laboratories #610124).

### shRNA-expressing virus

shRNAs targeting *Scop* was designed using siDirect (http://design.RNAi.jp/) and the following target sequence was used for the down regulation in mouse SCOP (GCATCACAGCGTATAAGCTCATTCAAGAGATGAGCTTATACGCTGTGATGC). A control shRNA with a scrambled sequence (GCTAACCTACTGACACGACTATTCAAGAGATAGTCGTGTCAGTAGGTTAGC) was designed. The pairs of the complementary oligonucleotides containing these sequences were synthesized (SIGMA), annealed and cloned into the modified FG12 lentivector, in which human pol III promoter was replaced by mouse pol III promoter. Original FG12 lentivector^57^ and helper plasmids were kindly provided by Xiao-Feng Qin (UT MD Anderson Cancer Center, Houston, TX).

HEK293T/17 cells were grown in DMEM (Nissui) with 10% FBS and plated on 10 cm dishes. After plating (80% confluent), a *Scop* shRNA expression plasmid and the helper plasmids were transfected using a transient calcium phosphate transfection method^58^. Eighteen hours after the transfection, the culture medium was replaced with the fresh medium (day 1). On day 2, the medium containing the first batch of the virus was collected, and was replaced with a fresh medium. This procedure was repeated on day 3, and the media collected on day 2 and 3 were combined. The virus-containing media was filtered through a 0.45 μm cellulose acetate membrane filter to remove cell debris, and then concentrated by ultracentrifugation at 50,000*g* for 90 min at 4 °C. The pellet was resuspended in 200 μl PBS and incubated for 30 min at 4 °C. The viral titre was quantified using HEK293T/17 cells by counting GFP-positive cells.

NIH3T3 cells were infected with shRNA expressing lentivirus and further cultured for 3 days before western blot analysis for SCOP protein expression.

### Stereotaxic injection of lentiviral vector

Mice (7 weeks old) were anaesthetized and mounted on a stereotaxic frame as described in the SCN-lesioning section. Lentivirus (1.5 × 10^8^ i.f.u.  ml^−1^) was injected bilaterally at two sites in each hemisphere (injection volume of 1.5 μl per site) into the hippocampal CA1 area at a rate of 0.1 μl min^−1^ using 30 G blunt-tip needle connected to a syringe pump by a polyethylene catheter. Two parallel injections were given to each unilateral CA1 region (that is, four injections per animal) for the infected area to cover the whole CA1 region, because a single injection of the lentiviral vector to each side of the CA1 region did not spread enough over the whole CA1. We set coordinates of the injection sites: AP −1.5 mm, ML ±0.8 mm and ±1.5 mm relative to bregma, and DV −1.5 mm for ML 0.8 mm and DV −1.3 for ML 1.5 mm relative to the skull surface^59^. After the injection, the needle was held in place for additional three minutes and then withdrawn.

### Preparation of membrane raft fraction

The raft fraction of the mouse hippocampus was prepared as previously described^38^ with modifications. Four pairs of mouse hippocampi were isolated for each data point with or without the dentate gyrus, and homogenized in buffer A (50 mM Tris-HCl, 1 mM EDTA, 2 mM EGTA, 50 mM NaCl, 10** **μg** **ml^−1^ aprotinin, 10 μg** **ml^−1^ leupeptin and 1 mM phenylmethanesulfonyl fluoride, pH 7.4) with a 10 × volume of the brain wet weight. The resulting homogenate was centrifuged at 1,000*g* for 15 min to sediment tissue debris. The unprecipitated material was centrifuged at 100,000*g* for 1 h. The resulting supernatant was collected as the ‘soluble fraction'. The membrane pellet was suspended in buffer A with 1% (v/v) Triton X-100 at a volume equal to that used for the initial homogenization, incubated for 1 h on ice and then combined with an equivalent volume of buffer A containing 80% (w/v) sucrose. Each sample was overlaid on buffer A containing 30% (w/v) sucrose (8 ml) and 5% (w/v) sucrose (2 ml), sequentially. After centrifugation at 100,000*g* for 18 h at 4 °C in a Beckman SW41 swing type rotor, the Triton X-100-insoluble cloudy materials floating at the interface between 5 and 30% sucrose solutions were collected as the raft fraction.

The raft fraction of the hippocampal primary neurons was prepared from 10 35 mm culture dishes combined. The cells were homogenized in 5 ml of buffer A and centrifuged at 100,000*g* for 1 h. The membrane pellet was suspended in 1 ml of buffer A with 1% (v/v) Triton X-100 and incubated for 1 h on ice, and then combined with an equivalent volume of buffer A containing 80% (w/v) sucrose. The sample was subjected to the discontinuous sucrose gradient as mentioned above in this section.

### Immunohistochemical analysis of SCOP

Mice were deeply anaesthetized by intraperitoneal injection of a mixture of ketamine and xylazine as mentioned above and intracardially perfused with saline containing 2 mM EDTA. The mice were then continuously perfused with ice-cold 2% paraformaldehyde in PBS. The brain was post-fixed in the same fixative for 3 h and then transferred into 30% sucrose in PBS overnight. The cryoprotected brains were frozen in dry ice and sliced into 20 μm-thick sections using a microtome-cryostat (Leica). The sections were washed three times with 0.3% Triton X-100 in PBS and incubated with 0.3% Triton X-100, 1% BSA, 1% normal goat serum in PBS for 4 h at 25 °C. They were then incubated with an anti-SCOP antibody, αEC (ref. 35) (1:50), overnight at 4 °C. The immunoreactivity was visualized with Alexa Fluor-conjugated donkey anti-rabbit IgG (1:1,000; Molecular Probes).

### Primary hippocampal neuron culture

Hippocampal neurons were cultured as previously described with some modifications^60^. Primary hippocampal neurons were cultured from newborn PER2::LUC mice^46^ and maintained in Neurobasal-A (minus phenol red) (GIBCO) with minimal supplements. Newborn pups were sacrificed by decapitation, and the hippocampi were digested in 5 ml of 10 units per ml papain at 37 °C for 30 min. After two rinses, the tissue was triturated three times in dissociation medium (27 mM K_2_SO_4_, 15 mM MgCl_2_, 74 mM Na_2_SO_4_, 18 mM glucose, 225 mM CaCl_2_, 0.0012% phenol red and 2 mM HEPES, pH7.4). Cells were plated onto 35 mm dishes coated with poly-D-lysine (BD Falcon) at 7.5 × 10^4^ cells per cm^2^ in growth medium (Neurobasal-A minus phenol red medium containing B-27 supplement (GIBCO), 100 U** **ml^−1^ penicillin, 100 μg^−1^ml^−1^ streptomycin and 0.5 mM glutamine). The medium was changed to the growth medium containing 5 μM arabinofuranosyl cytosine 24 h after plating. Neurons were maintained in the growth medium, and one-third of the medium was changed every 3 days. Plates were incubated in a 5% CO_2_ incubator at 37 °C. For the application of BDNF, 20 μl of 5 μg ml^−1^ BDNF (GIBCO) in 0.2% BSA or vehicle (0.2% BSA) was added to the medium at the final concentration of 50 ng ml^−1^ BDNF. Neurons were collected 15 min after the BDNF treatment. Neurons from 35 mm culture dishes were treated with 150 μl of SDS sample buffer and cells were scraped from the dish. The resulting homogenates were boiled for 3 min and subjected to SDS–PAGE and western analysis.

### Real-time monitoring of primary culture neurons

Real-time monitoring of cellular circadian gene expression was performed using primary culture neurons. At days *in vitro* 19, half of the medium was replaced with a recording medium (Neurobasal-A minus phenol red medium supplemented with B-27 supplement, 100 U ml^−1^ penicillin, 100 μg ml^−1^ streptomycin, 0.5 mM glutamine and 0.1 mM luciferin). The bioluminescence signals from the neurons were continually recorded at 37 °C under 5% of CO_2_ with dish-type bioluminescence detector, Kronos Dio (ATTO).

### RT–qPCR analysis

Total RNA was prepared from mouse hippocampus CA1 region using TRIzol reagent (Invitrogen) according to the manufacturer's protocol. RT–qPCR analysis was performed using Go Taq 2-step RT–PCR system (Promega). Data are presented as values normalized to the housekeeping gene *Rps29*. PCR primers used are; for *Bmal1* FW 5′-GCAGTGCCACTGACTACCAAGA-3′ and RV 5′-TCCTGGACATTGCATTGCAT-3′; for *Dbp* FW 5′-AATGACCTTTGAACCTGATCCCGCT-3′ and RV 5′-GCTCCAGTACTTCTCATCCTTCTGT-3′; for *Reverbα* FW 5′-CGTTCGCATCAATCGCAACC-3′ and RV 5′-GATGTGGAGTAGGTGAGGTC-3′; for *Rps29* FW 5′-TGAAGGCAAGATGGGTCAC-3′ and RV 5′-GCACATGTTCAGCCCGTATT-3′.

### Immunohistochemical analysis of p-ERK

Immunohistochemical analysis of p-ERK in the mouse CA1 was performed as described^61^ with slight modifications. Mice were cervically dislocated and the brains were rapidly isolated and cut into 2 mm coronal slices using a mouse brain matrix (RBM2000C, ASI Instrument). Within 2 min after the sacrifice, the slices were immersed in 4% paraformaldehyde/50 mM NaF/1 mM Na_3_VO_4_ in PBS, pH 7.4, overnight at 4 °C, cryoprotected in 30% sucrose and cut into serial coronal sections (20 μm) in a cryostat (Leica). Every third section between **−**1.58 and **−**2.46 mm relative to bregma was examined for immunoreactivity. The sections were pretreated with 0.3% Triton-X100 in PBS for 15 min, 50 mM boric acid buffer (pH 9.5) for 15 min and then 1.5% H_2_O_2_ in 10% ethanol in PBS for 20 min, blocked in 10% normal goat serum, 2% BSA, 50 mM glycine, 0.3% Triton-100 in PBS for 2 h and incubated in rabbit anti-phospho-p44/42 MAPK (1:5,000, Cell Signaling Technology, #9101). The signals were amplified by deposition of Alexa Fluor 488 tyramide complexes using Tyramide Signal Amplification System (PerkinElmer). The nine hippocampal sections per mouse were imaged on BZ-9000TS Microscope (Keyence). The images were binarized with a threshold at the average signal intensity of the oriens layer in each hippocampus using ImageJ software. The number of immuno-positive cells per CA1 pyramidal layer was integrated across the total 18 (9 bilateral) hippocampi in each animal. These numbers were averaged across animals in each group.

### Data availability

The authors declare that the data supporting the findings of this study are available within the article and its Supplementary Information files, or from the authors on request.

## Additional information

**How to cite this article:** Shimizu, K. *et al*. SCOP/PHLPP1β mediates circadian regulation of long-term recognition memory. *Nat. Commun.* **7,** 12926 doi: 10.1038/ncomms12926 (2016).

## Supplementary Material

Supplementary Information Supplementary Figures 1-7
