# Supplementary material for: SCOP/PHLPP1β mediates circadian regulation of long-term recognition memory
Source: Nat Commun. 2016 Sep 30;7:12926. doi: 10.1038/ncomms12926 (PMC5056436; doi:10.1038/ncomms12926)
Supplement: Supplementary Information — Supplementary Figures 1-7 [file ncomms12926-s1.pdf]

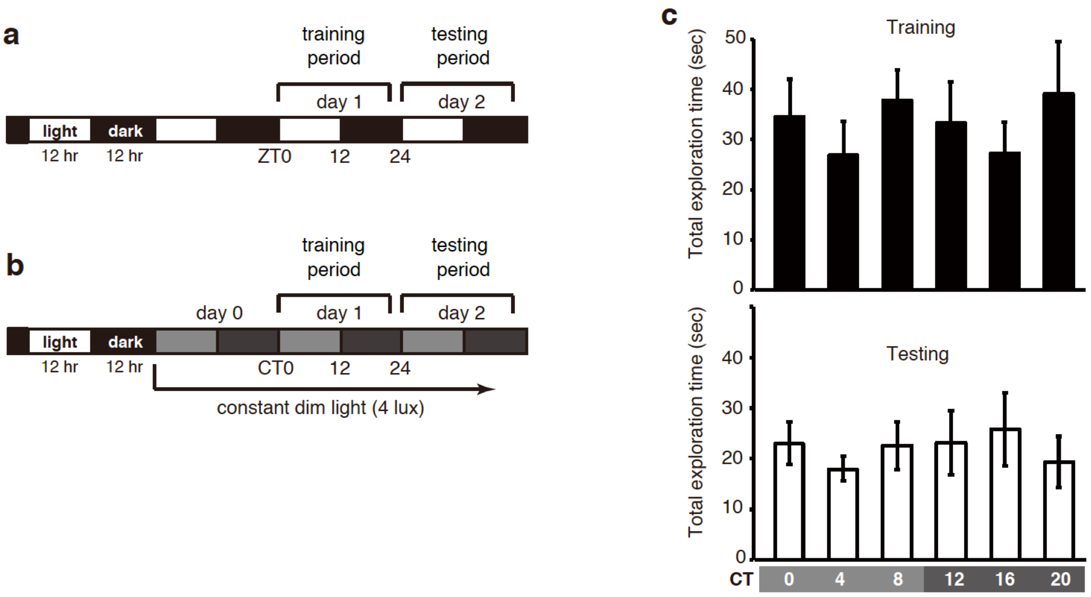

Supplementary Figure 1: Timelines of experiments on the novel object recognition task (a, b) and the total exploration time to objects (c).

(**a**) The timeline of our novel object recognition task under the LD cycle condition (Fig.1a). Mice were trained at various time points in day 1 and tested 24 hr after the training. (**b**) The timeline of the novel object recognition task under the constant dim light condition (Fig.1b). Mice were maintained in LD cycle for at least 2 weeks before starting the experiment. The mice were transferred to constant dim light condition (4 lux) at day 0. In this experiment, circadian time (CT) 0 was defined as 24 hr after the transfer of mice to the constant dim light condition. Mice were trained at various time points in day 1 and tested 24 hr after the training (Fig.1b). (**c**) Total exploration time to objects. In the experiments under constant dim light condition (Fig.1b), we observed no significant variation across the day in total exploration time to the objects during training (upper panel) or testing period (lower panel). p=0.92 (training; upper panel) and p=0.82 (testing; lower panel) by one-way ANOVA. Error bars, SEM (n=8)

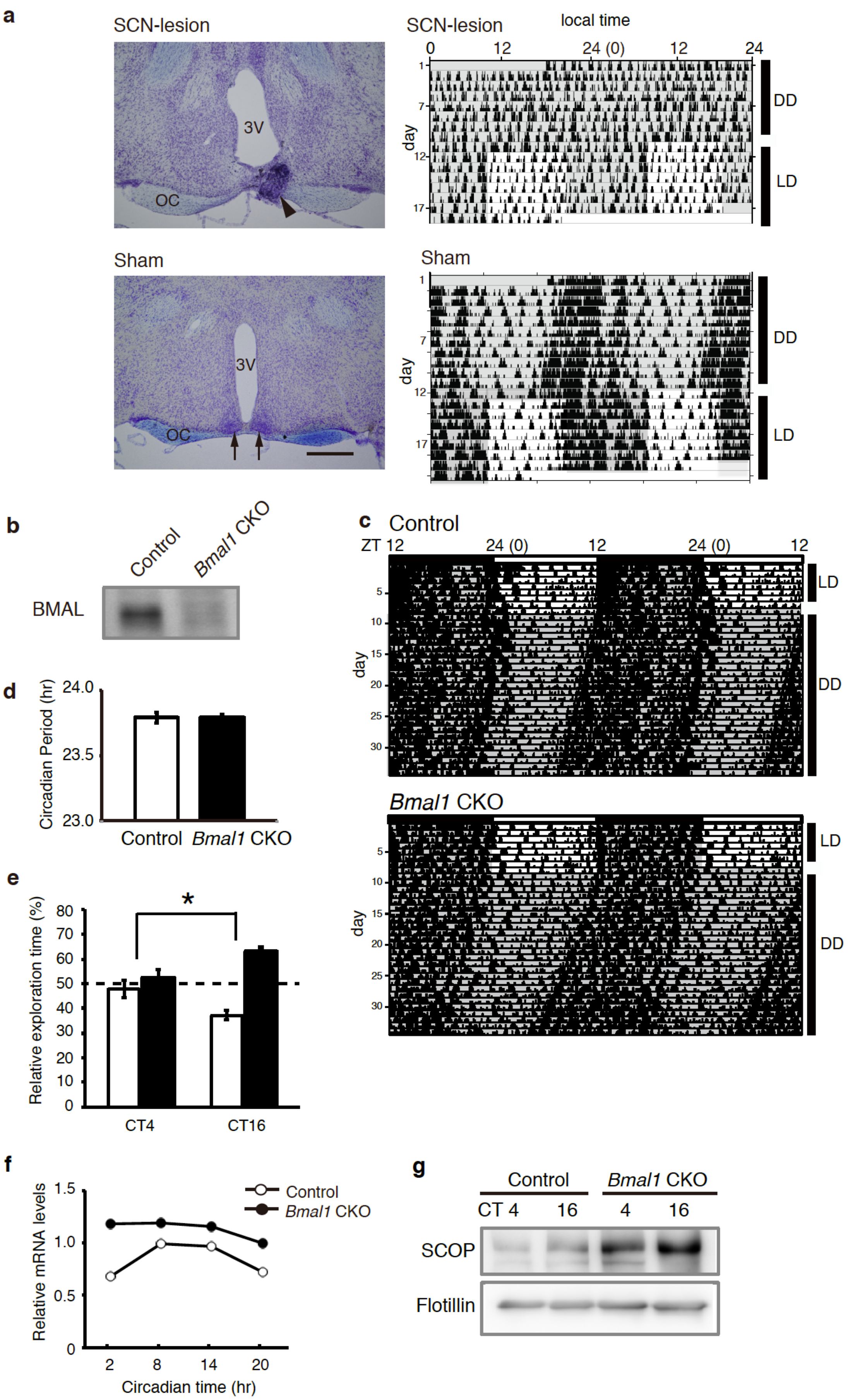
a
SCN-lesion
3V
OC
SCN-lesion
local time
0
12
24 (0)
12
24
day
1
7
12
17
DD
LD
Sham
3V
OC
Sham
1
7
12
17
DD
LD
b
Control
Bmal1 CKO
BMAL
c
Control
ZT 12
24 (0)
12
24 (0)
12
5
10
15
20
25
30
day
LD
DD
Bmal1 CKO
LD
DD
d
Circadian Period (hr)
24.0
23.5
23.0
Control
Bmal1 CKO
e
Relative exploration time (%)
80
70
60
50
40
30
20
10
0
*
CT4
CT16
f
Relative mRNA levels
1.5
1.0
0.5
0
Control
Bmal1 CKO
2
8
14
20
Circadian time (hr)
g
Control
Bmal1 CKO
CT 4
16
4
16
SCOP
Flotillin

Supplementary Figure 2: Ablation of the SCN clock and the forebrain clock.

(**a**) Shown are the representative Nissl-stained coronal brain sections (left panels) and double-plotted actograms (right panels) of mice receiving an electrolytic lesion to the SCN (upper panels) and sham operation (lower panels). The intact bilateral SCN (arrows) in the section of sham-operated mouse is shown, while SCN is lost in the section of SCN-lesioned mouse. Tissue debris (arrowhead) is yielded frequently by the electrical lesioning. 3V, third ventricle. OC, optic chiasm. Scale bar, 500 µm. Locomotor activities of LD-entrained mice were recorded under constant dark condition for 10 - 12 days, and then the mice were transferred to the LD cycle. Shaded areas on the actograms signify the dark period. (**b**) Western blot analysis for BMAL1 protein prepared from the nucleus fraction of forebrain of *Bmal1* CKO (*Bmal1*$^{flox/flox}$;*Emx1*$^{cre/+}$) and control (*Bmal1*$^{flox/flox}$;*Emx1*$^{+/+}$) mice. (**c**) A representative double-plotted actograms of *Bmal1* CKO mice and littermate control mice. Locomotor activity was recorded initially in LD condition for 8 days, and then recorded in DD condition. Shaded areas on the actogram signify the dark period. (**d**) Circadian periods of free-running activities in DD determined by chi-square periodgram. Error bars, SEM (n=10). (**e**) Long-term memory performance of *Emx1*$^{cre/+}$ mice in the novel object recognition task. * p=0.014 by Student's t-test. (**f**) Temporal changes in *Scop* mRNA levels in the hippocampal CA1 of *Bmal1* CKO (*Bmal1*$^{flox/flox}$;*Emx1*$^{cre/+}$) or control mice (*Bmal1*$^{flox/flox}$;*Emx1*$^{+/+}$). mRNA levels were measured by real-time RT-PCR. Three pairs of hippocampi were pooled and used at each time point. (**g**) Western blot analysis for SCOP protein in the membrane rafts of hippocampal CA1 in *Bmal1* CKO and control mice at CT4 and CT16. Three pairs of hippocampi were pooled and used in each group.

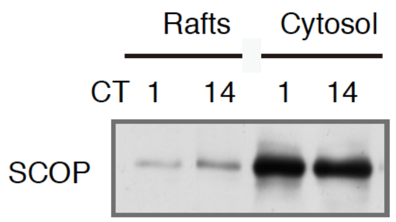

Supplementary Figure 3: Western blot analysis of SCOP proteins in the membrane raft and cytosolic fractions prepared from the hippocampal CA1 at CT1 or CT14. One-twentieth volume of the whole raft fraction or one-eightieth of the cytosolic fraction was loaded in each lane.

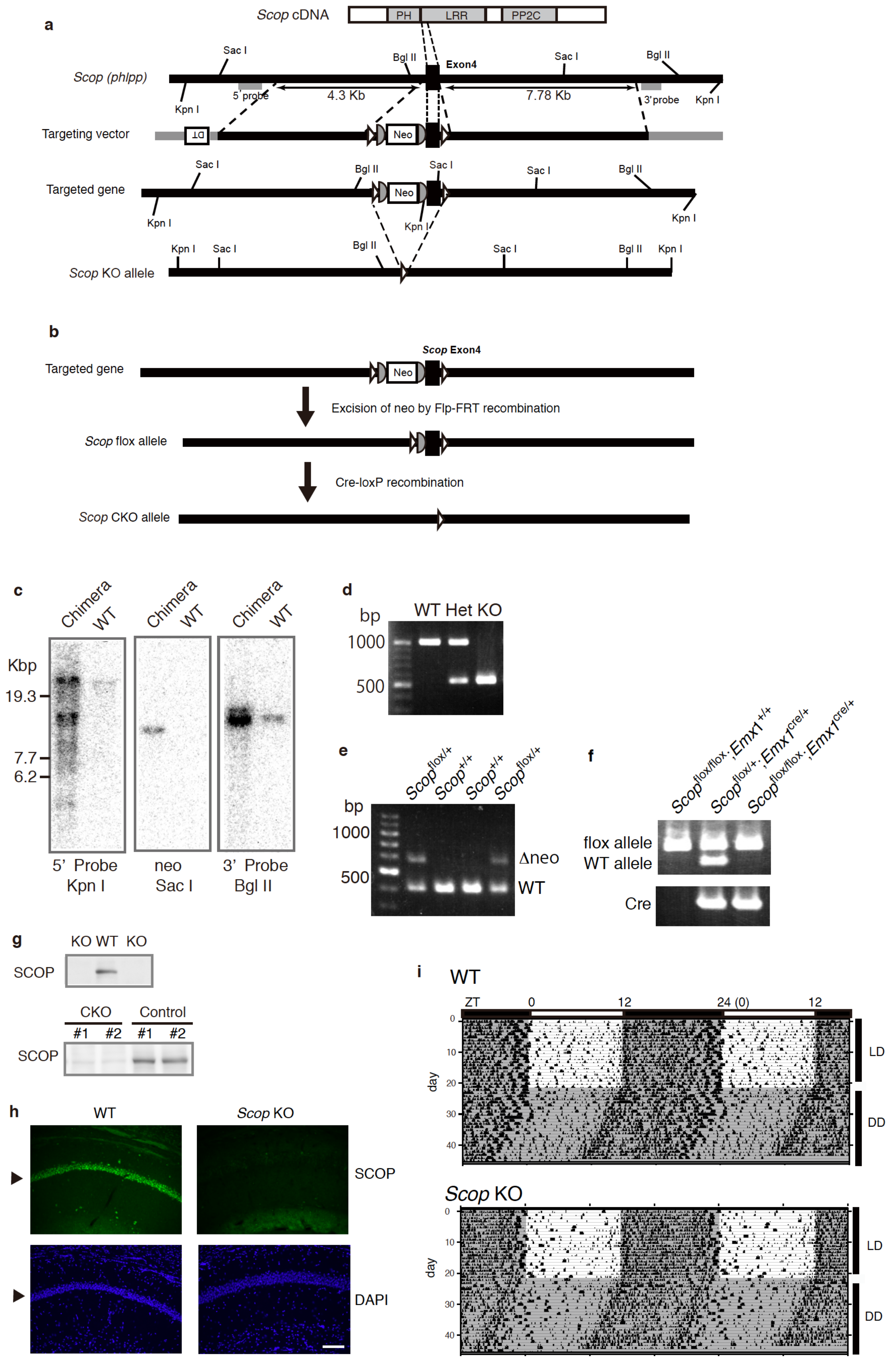

a
Scop cDNA
PH
LRR
PP2C
Scop (phlpp)
Sac I
Bgl II
Exon4
Kpn I
5' probe
4.3 Kb
7.78 Kb
3' probe
Targeting vector
DT
Neo
Targeted gene
Scop KO allele
b
Scop Exon4
Targeted gene
Excision of neo by Flp-FRT recombination
Scop flox allele
Cre-loxP recombination
Scop CKO allele
c
Chimera
WT
Kbp
19.3
7.7
6.2
5' Probe
Kpn I
neo
Sac I
3' Probe
Bgl II
d
bp
WT Het KO
1000
500
e
Scop flox/+
Scop +/+
Scop +/+
Scop flox/+
bp
1000
500
Δneo
WT
f
Scop flox/flox ; Emx1 +/+
Scop flox/+ ; Emx1 cre/+
Scop flox/flox ; Emx1 cre/+
flox allele
WT allele
Cre
g
KO WT KO
SCOP
CKO
Control
#1 #2 #1 #2
SCOP
h
WT
Scop KO
SCOP
DAPI
i
WT
ZT
0
12
24 (0)
12
day
0
10
20
30
40
LD
DD
Scop KO
0
10
20
30
40
day
LD
DD

Supplementary Figure 4: Generation of *Scop* KO mouse and its behavioral rhythm.

(**a**) Generation of *Scop* KO mice using the Cre/loxP recombination system. Schematic representation of cDNA, WT allele, targeting vector, targeted gene, and KO allele after Cre-mediated recombination. Solid boxes represent exon4. Open boxes represent neomycin (Neo) and diphtheria toxin (DT) cassettes. *LoxP* sites are indicated by open triangles. *frt* sites are indicated by gray semicircles. Gray bars indicate the probe regions used in Southern blot analysis. (**b**) Generation of forebrain-specific *Scop* conditional knockout mice. The FRT-flanked neo gene was excised via Flp-FRT recombination, and floxed exon4 was deleted via Cre-loxP recombination by using *Emx1*-Cre mice. (**c**) Southern blot analysis for genomic DNAs from chimeric and WT mice. (**d**) PCR genotyping of offsprings from intercrossing *Scop* KO mice. A 1.0-kb WT band and a 0.5-kb mutant band were amplified with the primer set targeting the *Scop* gene. (**e**) PCR genotyping of Δneo flox allele. (**f**) PCR genotyping of $Scop^{flox/flox}$;$Emx1^{cre/+}$ (*Scop* CKO). (**g**) Western blot analysis for SCOP protein prepared from the hippocampus of WT and *Scop* KO (upper panel) and *Scop* CKO ($Scop^{flox/flox}$; $Emx1^{cre/+}$) and control ($Scop^{flox/flox}$; $Emx1^{+/+}$) mice (lower pannel). (**h**) Coronal sections of the hippocampus were immuno-stained with SCOP antibody (αEC). SCOP immunoreactivities were absent in the hippocampal CA1 pyramidal cell layer of *Scop* KO. Arrows indicate CA1 pyramidal cell layers. Scale bar represents 100 μm. (**i**) A representative double-plotted actograms of WT and *Scop* KO mice. Locomotor activities were recorded initially in LD condition for 22 days, and then recorded in DD condition. Shaded areas on the actograms signify the dark period.

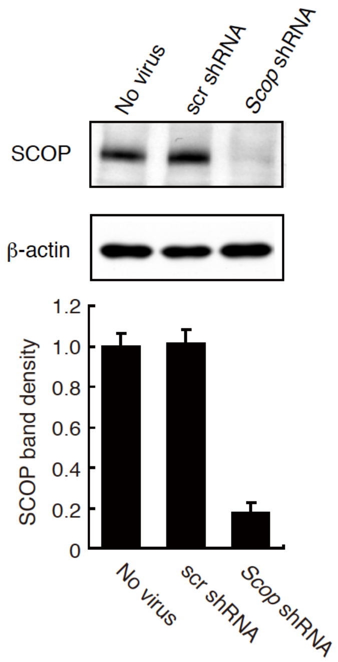

Supplementary Figure 5: Evaluation of knockdown activity of anti-*Scop* shRNA lentivirus.

Decrease in SCOP protein level in NIH3T3 cells by infection of anti-*Scop* shRNA lentivirus (*Scop* shRNA). Scrambled shRNA lentivirus (scr shRNA) was used as a control. Representative western blot data of SCOP protein and β-actin in NIH3T3 cells are shown. The bar graph shows averaged relative SCOP protein levels. Error bars, SEM (n = 3)

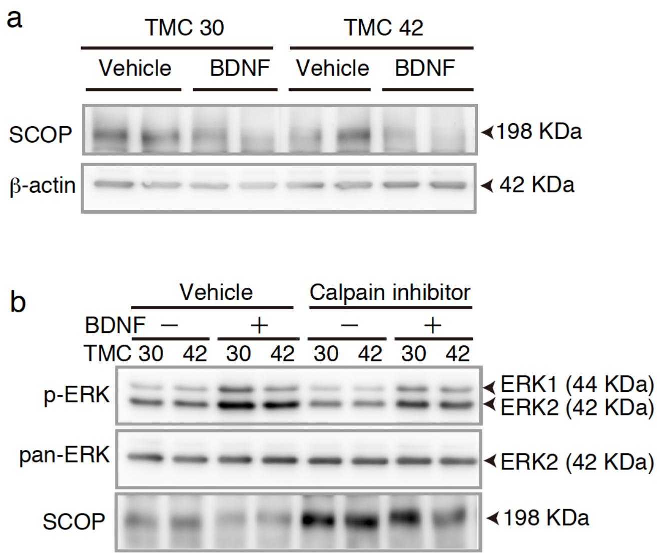

Supplementary Figure 6: BDNF-induced degradation of SCOP (a) and BDNF-induced ERKs activation sensitive to calpain inhibitor (b) in hippocampal primary culture neurons.

(**a**) Western blot analysis for SCOP protein in total lysate prepared from hippocampal primary culture neurons of PER2::LUC knock-in mice. Each lane represents a different individual dish. (**b**) Western blot analysis for p-ERKs and SCOP protein in total lysate prepared from hippocampal primary culture neurons of WT mice. BDNF treatment induced ERKs activation and degradation of SCOP protein at TMC30 and 42. Forty-minutes application of calpain inhibitor III (final, 25μM) suppressed the degradation of SCOP (bottom panel), and the more potent BDNF-induced ERK activation at TMC30 than that at TMC42 (top panel, 3rd and 4th lanes) was markedly reduced by the inhibitor treatment (7th and 8th lanes). Similar results were observed from n=3 biological replicates.

Fig. 3a SCOP in rafts

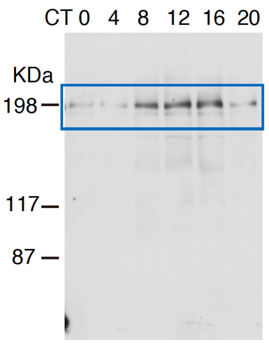

Fig. 3a K-Ras & Flotillin

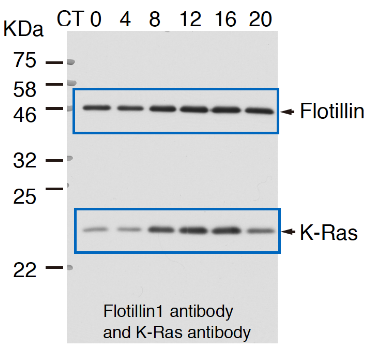

Fig. 3a SCOP in cytosol

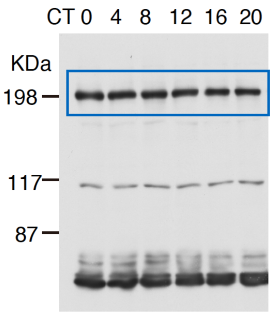

Fig.3a β-actin

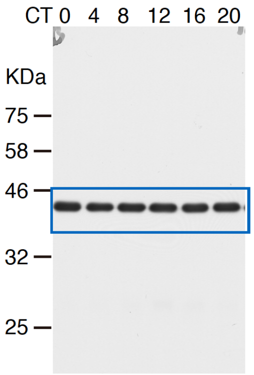

Fig.3b SCOP in rafts

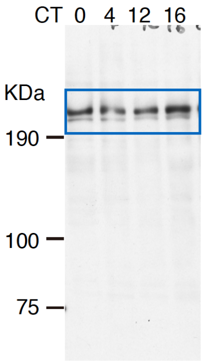

Fig.3b K-Ras & Flotillin

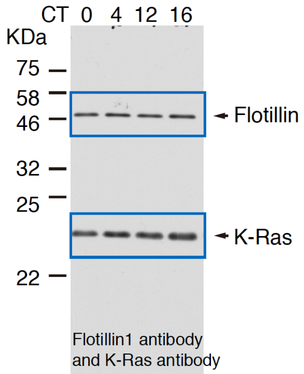

Fig.3b SCOP in cytosol

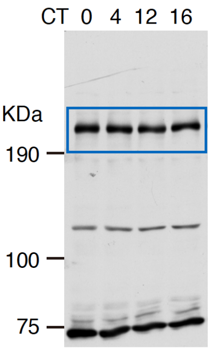

Fig. 3c SCOP

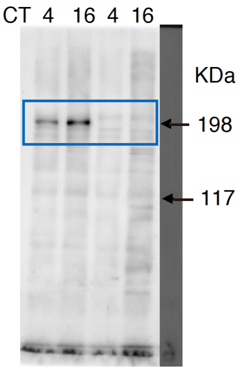

Fig. 3c K-Ras & Flotillin

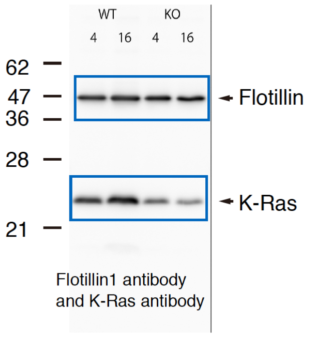

Fig. 5c SCOP

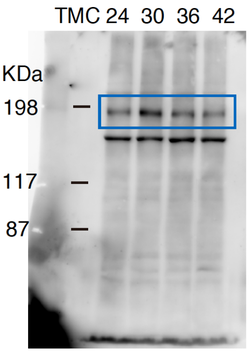

Fig. 5c SCOP

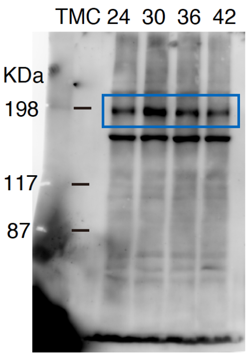

long-exposure

Fig.5c Flotillin

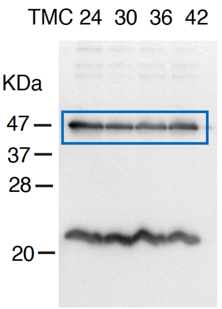

Fig. 5d p-ERK

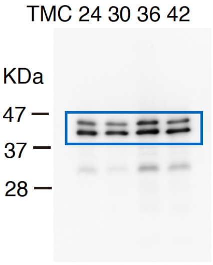

Fig. 5d pan-ERK

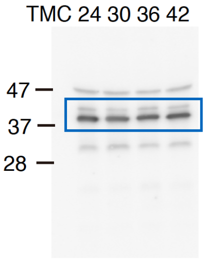

Fig. 5e p-ERK

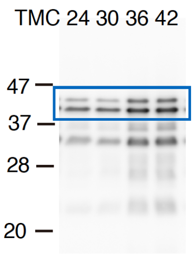

Fig.5e pan-ERK

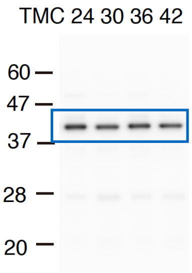

Supplementary Figure 2b

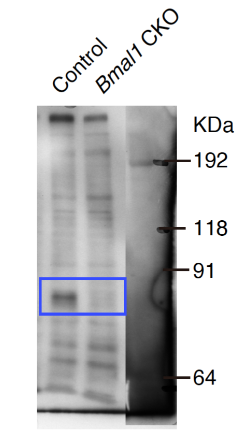

Supplementary Figure 2g (SCOP)

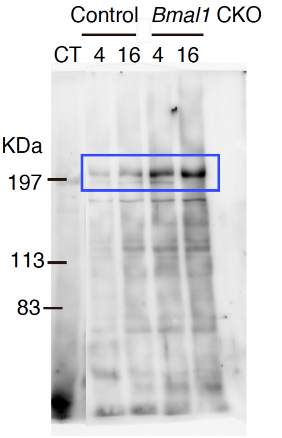

Supplementary Figure 2g (Flotillin)

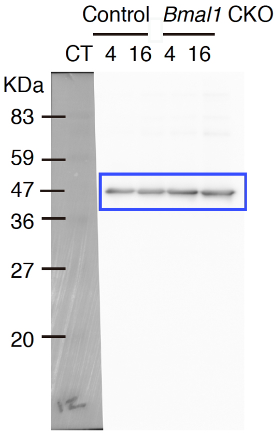

Supplementary Figure 3

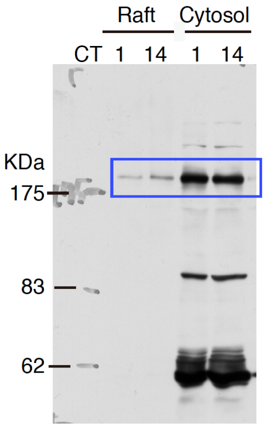

Supplementary Figure 4g

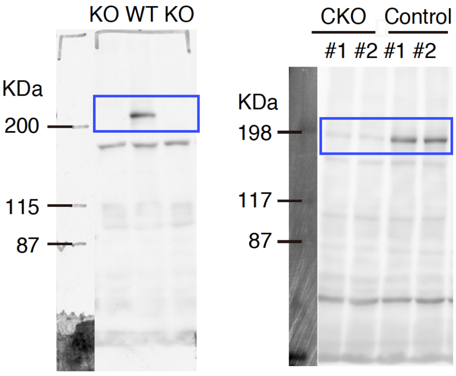

Supplemental Figure 5 (SCOP)

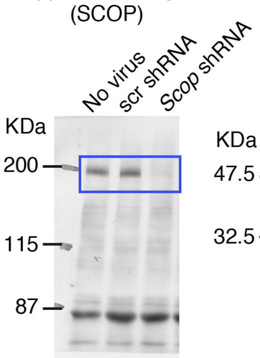

Supplemental Figure 5 (β-actin)

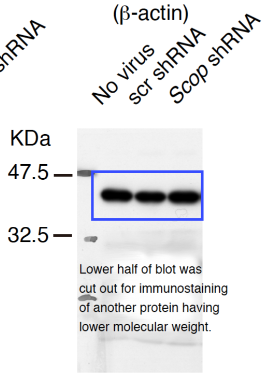

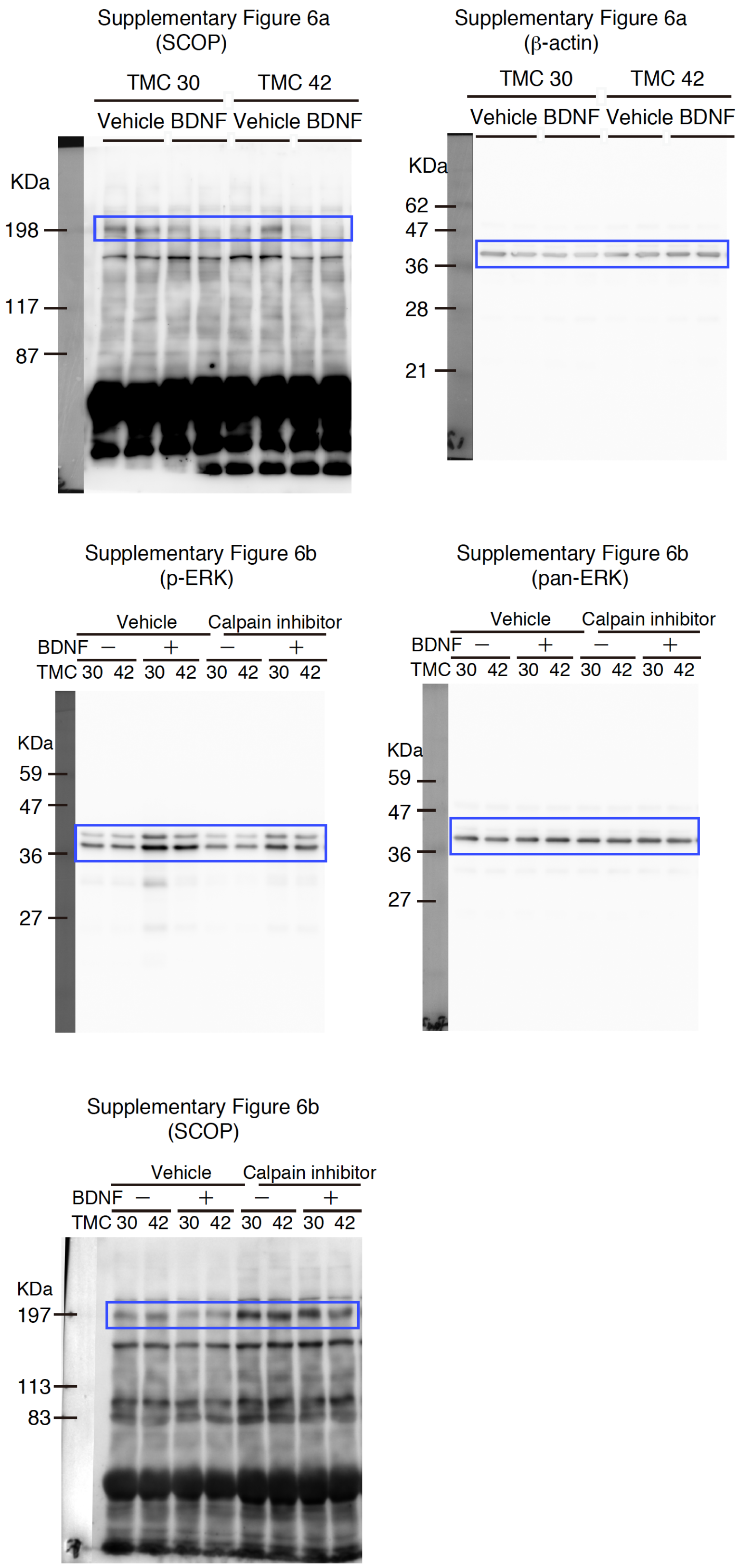

Supplementary Figure 7: Full images of all the western blots data shown in the manuscript.
